# Case report: Temporal alterations in vascular function during the first 2 weeks of pediatric septic shock

**DOI:** 10.3389/fped.2022.939886

**Published:** 2022-07-22

**Authors:** Christiaan Diederik Mathijs Wijers, Ryan J. Stark

**Affiliations:** ^1^Department of Pathology, Microbiology, and Immunology, Vanderbilt University Medical Center, Nashville, TN, United States; ^2^Department of Pediatric Critical Care, Vanderbilt University Medical Center, Nashville, TN, United States

**Keywords:** pediatric, sepsis, vascular function, shock, LDPMI

## Abstract

**Introduction:**

During sepsis and septic shock, the host's immune systems generate an overwhelming and often, detrimental, inflammatory response. Part of this response results in significant alterations in blood flow and vasomotor tone regulated in part by endothelial and vascular smooth muscle cells. Here, we report on a series of 3 pediatric patients for whom vascular response was assessed by laser doppler perfusion coupled to iontophoresis over the first 2 weeks after hospitalization for septic shock to demonstrate similarities and dissimilarities in the vascular response.

**Case Presentations:**

A 12-year-old male with a history of Burkitt's Lymphoma, a 21-year-old male with congenital porencephaly and epilepsy, and a 7-year-old male with no significant past medical history all were admitted to a tertiary care children's hospital with a diagnosis of septic shock requiring vasoactive infusions to maintain mean arterial blood pressure. Non-invasive laser doppler perfusion coupled with iontophoresis of either acetylcholine (endothelial-dependent response) or sodium nitroprusside (endothelial-independent response) was performed on hospital days 1, 3, 7, and 14. Variability and heterogeneity were demonstrated by the temporal assessments of the vascular response to sodium nitroprusside, but all three patients showed significant similarity in the temporal responsiveness to acetylcholine.

**Conclusion:**

Assessment of baseline and temporal responsiveness to endothelial-dependent vascular reactivity may provide a predictable timeline to the resolution of pediatric septic shock.

## Introduction

Pediatric sepsis and septic shock remain a significant contributor to morbidity and mortality worldwide (1). A deranged and detrimental host response to pathogens leads to the many classical features of sepsis such as impaired mental status, increased oxygen demand, and alterations in cardiac output (CO). Underlying these features are changes within the vasculature, designed to augment blood flow to the areas most in need while shunting blood away from areas that can temporarily be sacrificed (2). These alterations in blood flow are dependent on the surrounding inflammatory milieu and the response of the vasculature to internal and external mediators that regulate vascular tone. For the endothelial cells, the cells that line every blood vessel in the body, generation of endogenous nitric oxide (NO) causes vasomotor relaxation of adjacent smooth muscle cells (3). While endothelial cells are one source, NO can also be produced by other sources such as white blood cells, which bypass the endothelium and directly act on the vascular smooth muscle. The end result of either process is NO activation of cyclic-guanosine 3',5'-monophosphate (cGMP) leading to calcium movement into the sarcoplasmic reticulum and thus, relaxation (4).

Assessment of these physiologic responses can be determined clinically utilizing laser doppler perfusion monitoring coupled with iontophoresis (LDPMI) of the respective endothelial-dependent or -independent pharmacologic agents. In these conditions, acetylcholine (ACh) can be delivered trans-dermally to induce endothelial-dependent relaxation and be compared to sodium nitroprusside (SNP) which can induce an endothelial-independent response. Vascular responses utilizing these techniques have been shown to correlate with the severity of illness as measured by pediatric sequential organ failure assessment scores (5). However, the host response to sepsis is dynamic and heterogenic and thus the question is raised as to how similar or dissimilar are these responses during the temporal course of septic shock and its resolution. Herein, we describe the vascular response to ACh and SNP with LDPMI utilizing a previously established protocol between three diverse cases of septic shock admitted to a tertiary children's hospital during the first 2 weeks of hospitalization.

## Case presentations

### Case 1

**Figure 1:**
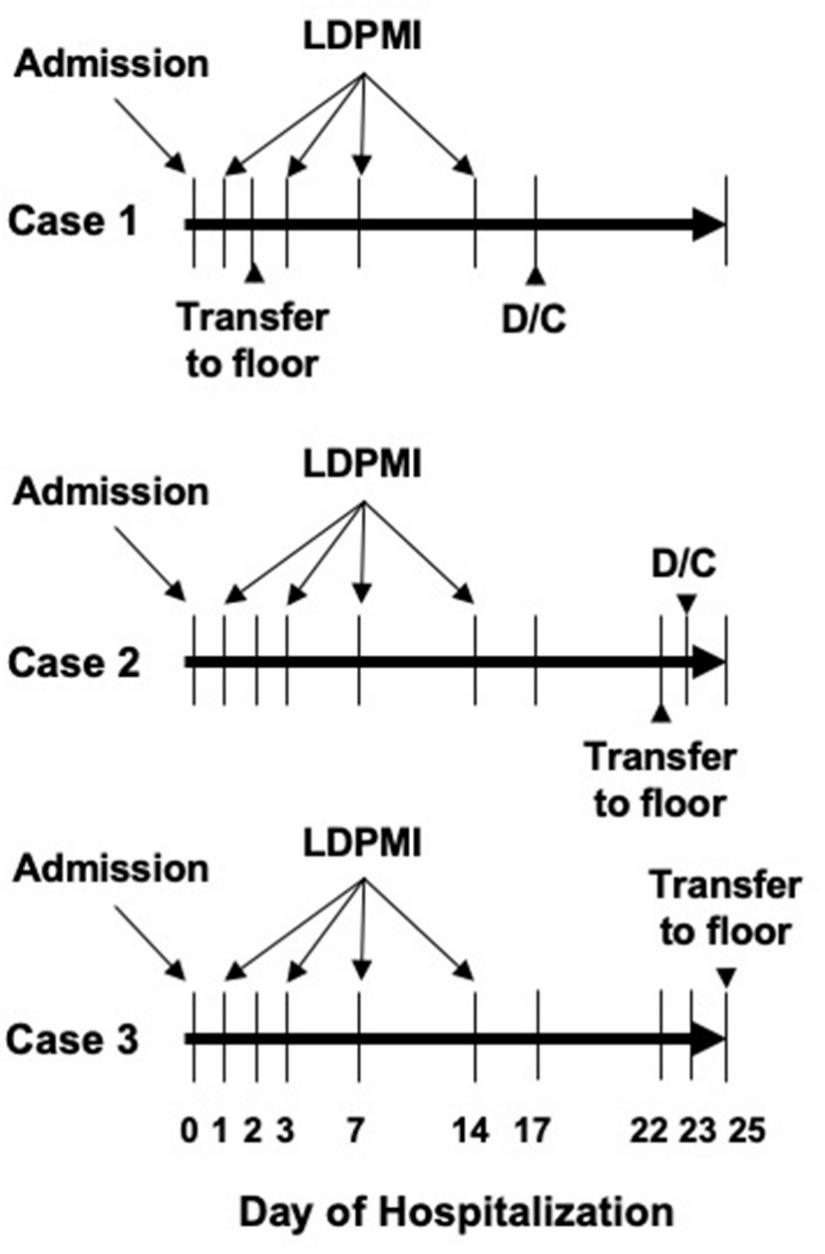
Schematic of basic hospital course for each patient included in this series. Days of admission, transfer to general pediatric ward, and discharge (D/C) are shown. LDPMI measurements were taken on days 1, 3, 7, and 14 for each patient.

**Figure 2:**
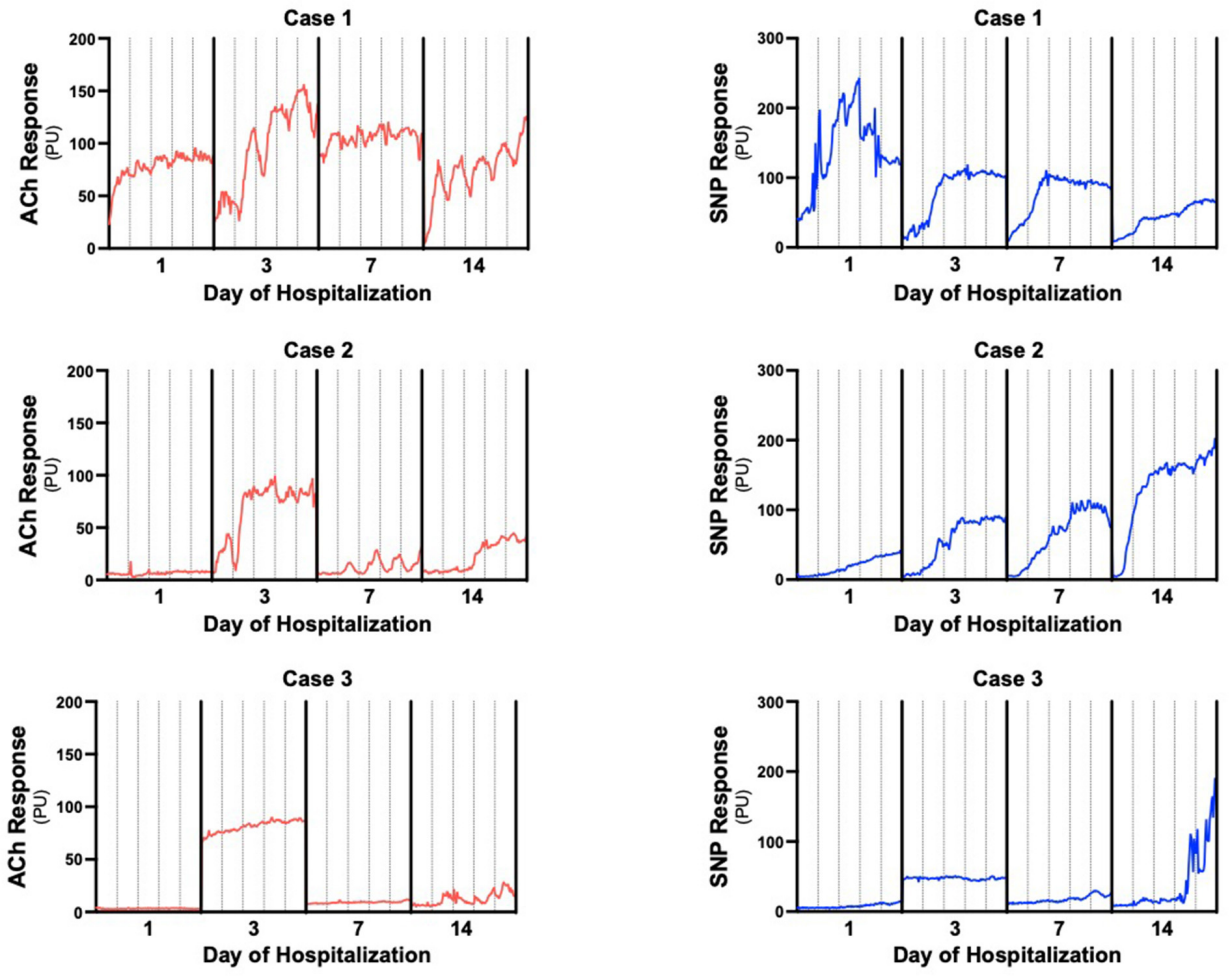
Individual LDPMI vascular response recordings for each patient. Measurements were collected at 0.03 s intervals then displayed as averaged perfusion units every 5 s to generate the plots to reduce movement artifacts. Acetylcholine (ACh) responses are in red, sodium nitroprusside (SNP) responses are in blue. Solid vertical lines separate days of measurement, dashed vertical lines are 2 min intervals to estimate individual iontophoresis pulses.

**Figure 3:**
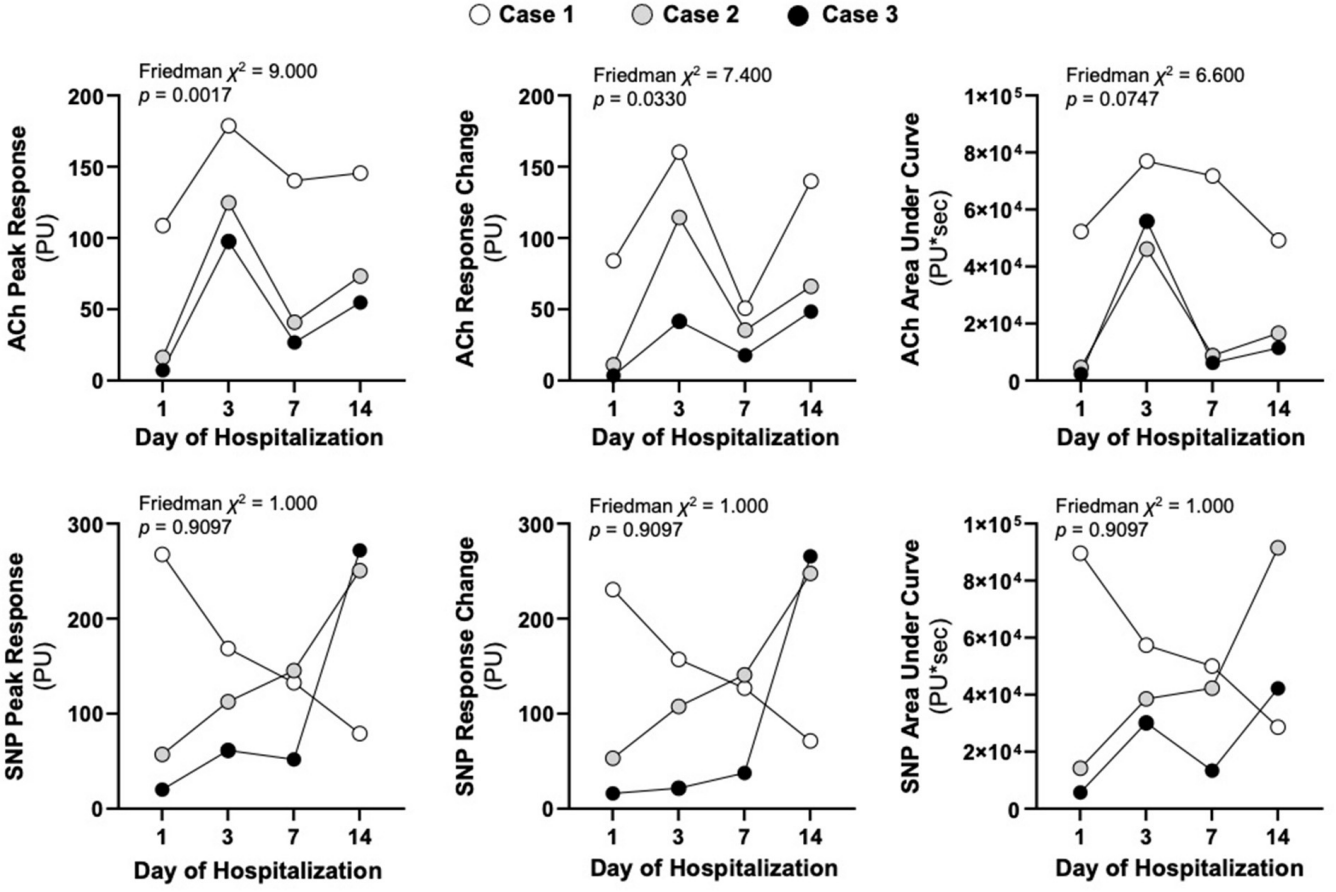
Respective LDPMI assessments to acetylcholine (ACh) and sodium nitroprusside (SNP) for each patient on the stated day of hospitalization. The peak response (maximal perfusion), the response change (the change in baseline perfusion to peak response) and the area under the curve (cumulative dose response) are shown at each of the time points. Friedman chi-squared tests were used to assess similarity of non-parametric repeated measurements across the three patients.

The patient was a 12-year-old male with a history of Burkitt's Lymphoma currently undergoing maintenance chemotherapy who presented in septic shock after a 1 day history of fevers and chills. He initially required both epinephrine and norepinephrine, but these were able to be quickly weaned off and he was on no vasoactive infusions at the time of first LDPMI measurement on Day 1. LDPMI measurement was performed as previously described (6). Briefly, Laser Doppler perfusion monitoring (PeriFlux 5010, Perimed, Stockholm, Sweden) was coupled with iontophoresis (Perilont 382b, Perimed) of vasoactive compounds. The drug delivery electrode with the LDPM probe was soaked with 180 microliters of 2% ACh (Sigma) and secured to the volar aspect of the patient's distal forearm. A dispersive electrode was attached 15 cm distal to the drug delivery electrode on the proximal forearm. Prior to the start of iontophoresis, basal perfusion was recorded for 2 min. ACh was then delivered with a 0.1 mA anodal current for 20 s for a total of 5 doses separated by 60 s. Perfusion was monitored for a total of 10 min. The protocol was repeated on the opposite arm using 180 microliters of 1% sodium nitroprusside (SNP, Sigma) and a 0.2 mA cathodal current. Measurements were repeated on Days 1, 3, 7, and 14 using the same protocol and measurement sites (Figure 1). Raw LDPMI measurements, reported in perfusion units (PUs), for each patient are provided in Figure 2. Comparisons for peak response, change in response and area under the curve are shown in Figure 3 and were assessed for similarity among patients using a Friedman Chi-squared test with Dunn's multiple comparisons.

The patient's initial lactate on admission was 0.8 mmol/L and his white blood cell (WBC) count on Day 1 was 2.8 × 10^3^/mcL. His microbiology cultures were subsequently positive for *E. cloacae* bacteremia. He did require high flow nasal cannula on Day 1 but this was weaned off by Day 2 and he was subsequently transferred to the general pediatric unit to complete ongoing chemotherapy. He remained hemodynamically stable during LDPMI measurements on Days 3, 7, and 14. His WBC counts on Days 3, 7, and 14 were 4.2, 7.8, and 1.8 (×10^3^/mcL), respectively. He was discharged in his baseline state of health on Day 17.

### Case 2

The patient was a 21-year-old male with a past medical history significant for congenital porencephaly, spastic quadriplegia and epilepsy with associated developmental delay who presented with several days of congestion and cough with a 1 day history of epistaxis and respiratory distress. He was intubated on presentation and required mechanical ventilation as well as vasoactive support with epinephrine (0.16 mcg/kg/min) and norepinephrine (0.09 mcg/kg/min). He had a lactate of 5.0 mmol/L and a WBC count of 4.9 × 10^3^/mcL at the time of LDPMI measurements on Day 1. His respiratory cultures were positive for *P. aeruginosa, S. agalactiae* and *S. aureus*. By Day 3, he was continuing to require norepinephrine (0.02 mcg/kg/min) in addition to mechanical ventilation. His WBC count was 6.1 × 10^3^/mcL. By Day 7, the patient remained on mechanical ventilation and had been transitioned to epinephrine (0.04 mcg/kg/min) and isoproterenol (2 mcg/min) for bradycardia. His WBC count was 8.1 × 10^3^/mcL. By Day 14, the patient had been extubated to non-invasive bilevel ventilation after 12 days of invasive mechanical ventilation and was no longer requiring vasoactive support. His WBC count was 14.3 × 10^3^/mcL. He remained in the intensive care unit till Day 22 and was discharged from the general pediatric unit on Day 23 with new home nighttime non-invasive ventilation.

### Case 3

The patient was a 7-year-old male with no known past medical history who presented in septic shock after a 1 week history of malaise with a 2 day history of headache and vomiting. The patient was quickly escalated on vasoactive medications and required mechanical ventilation. At the time of LDPMI measurements on Day 1, the patient was on epinephrine (0.22 mcg/kg/min), norepinephrine (0.22 mcg/kg/min) and vasopressin (0.04 units/kg/min) as well as venoarterial extracorporeal membrane oxygenation (VA-ECMO, flow 4.5 L/min) for vasoactive refractory shock. His lactate at this time was 11.5 mmol/L and his WBC count was 29.3 × 10^3^/mcL. His subsequent cultures were positive for Influenza A, Norovirus and *S. pyogenes* bacteremia. By Day 3, his WBC count was 22 × 10^3^/mcL and the patient was continued on VA-ECMO (flow 4.59 L/min) as well as receiving renal replacement therapy and plasma exchange. The patient still required epinephrine (0.14 mcg/kg/min) and norepinephrine (0.14 mcg/kg/min). By Day 7, the patient had been decannulated from ECMO after a 6 day course but continued to require mechanical ventilation with epinephrine support (0.02 mcg/kg/min). His WBC count was 26.3 × 10^3^/mcL. The patient developed *C. glabrata* fungemia on Day 11 and his central line was removed. By hospital Day 14, the patient had been extubated after 9 days total of mechanical ventilation and was no longer requiring vasoactives, but remained on intermittent hemodialysis. His WBC at this time was 13.6 × 10^3^/mcL. He was transferred to the general pediatric unit on Day 25 and his hemodialysis was discontinued on Day 27 with improvement in his native renal function, though he required antihypertensive agents (losartan, atenolol and clonidine) for blood pressure control. He was transferred to an inpatient pediatric rehabilitation facility on Day 52.

Raw LDPMI measurements for each patient (Figure 2) and comparisons across patients (Figure 3) are shown. Endothelial-dependent ACh peak response, ACh response change, and ACh area under the curve were relatively homogeneous across the three patients included in this series, despite their dissimilar presentations and course (Figures 2, 3). Specifically, ACh peak response and response change were consistently higher or lower in a time-dependent manner across the three patients included in this series (Friedman chi-squared test: *p* = 0.0017 and *p* = 0.0330, respectively). Visually, this can be appreciated from the individual recordings of the vascular response to ACh. By contrast, endothelial-independent SNP peak response, SNP response change, and SNP area under the curve were more dissimilar than not across the three patients over time.

## Discussion

As part of the adaptive inflammatory response to infection, the vascular endothelium is activated in response to cytokines and pathogen associated molecular patterns. This leads to changes in vasomotor tone and increased blood flow to the infected area and vital organs (7–9). However, in the setting of severe sepsis and septic shock, both the endothelium and the vascular smooth muscle become dysfunctional, resulting in impaired vascular reactivity and altered regional blood flow (2, 7, 8, 10). In this case series, despite patient heterogeneity, we found that endothelial responsiveness was quite similar in terms of temporal variation across the different timepoints, while responsiveness of the vascular smooth muscle was not. Though these are preliminary findings, they implicate a role for the vascular endothelium that is rather homogenous in its temporal responsiveness to severe infections, while more heterogeneity exists within the smooth muscle response. The lack of predictability in vascular responses to SNP was consistent with prior observations. In one study, vasodilation in response to ACh was significantly attenuated in skeletal muscle feed arteries obtained from older subjects in comparison to those from younger subjects, while no differences in vasodilation in response to SNP were found (11). The pathophysiological mechanism for this observation is likely multifactorial and merits further inquiry. One potential contributing factor might be that endothelial-independent mechanisms, which are reflected by vascular responses to SNP, are more susceptible to external sources of nitric oxide (NO) such as WBCs. Prior observations have shown that there exists an inversely proportional relationship between WBC counts and vascular responsiveness to SNP (12, 13). What effect external sources of NO contributed to the data obtained in this series is unclear, but it is worth noting that the patient with the lowest WBC count by Day 14 (Case 1) had much lower SNP responses compared to the patients with more elevated WBC counts by the same day of hospitalization (Cases 2 and 3). Interestingly, the WBC count and SNP responses did appear more inversely proportional in the acute phase of illness on Day 1, but more directly proportional by Day 14. Whatever the precise impact of these findings, they suggest that LDPMI-based assessment of vascular responsiveness may have the potential to offer insights into clinical outcomes during and after pediatric sepsis.

Clinical assessment of physiologic aberrations that are non-invasive are sorely lacking in critical illness. Herein, we describe the vascular responses to ACh and SNP (pharmacological provocation) using non-invasive LDPMI to gauge a sense of temporal alterations within vascular physiology over the course of a disease. The use of LDPM to assess patient outcomes, particularly in the setting of sepsis, has been previously performed in adult populations. In one study, the investigators compared vascular responsiveness to ACh as measured by LDPM in two separate anatomical locations: the knee and the forearm (14). Relative to survivor patients with septic shock, it was found that endothelial dysfunction in the knee was significantly greater in non-survivors. Alternatively, there were no statistically significant differences in endothelial dysfunction in the forearm between these two patient populations (14). While the exact reason for the difference in endothelial dysfunction between the two anatomical sites remains unclear, spatial heterogeneity in terms of endothelial dysfunction, as has been described in the gastrointestinal tract in an animal model of sepsis, may be one reason (15). In addition, differences between the knee and forearm in terms of tissue depth and vascular density constitute another potential explanation. However, it should be noted that in a majority of studies published using LDPM in sepsis, the most commonly used anatomical location for assessing vascular responsiveness is the forearm (7). Irrespective, while there are likely a multitude of biomechanical reasons for the observed differences, it has become increasingly evident that ACh responsiveness appears to be a better indicator of vascular dysfunction and severity of disease in pediatric critical illness compared to those mediated by SNP (5, 6).

Outside LDPM, other non-invasive techniques to assess vascular responses to pharmacological and/or ischemic provocation include venous occlusion plethysmography and flow-mediated brachial artery vasodilatation measured by ultrasound (7). Venous occlusion plethysmography provides a measure of blood flow to a specific anatomical site between two cuffs using electrodes (16), whereas brachial artery vasodilation measured by ultrasound is used to determine the maximum brachial artery diameter following occlusion pressure release (17). Additionally, reactive hyperemia peripheral arterial tonometry (RH-PAT) can be used to assess vascular responsiveness. Specifically, peripheral artery pulse amplitude is measured using RH-PAT probes following the release of an occluding pressure (18). Echocardiography may also be used to non-invasively monitor CO. While this method does not measure vascular reactivity, it can help determine fluid responsiveness, myocardial function, and obstructive physiology (19, 20). In children with septic shock, myocardial dysfunction is common, and echocardiography can aid in its early recognition (19–21). Similarly, an ultrasonic cardiac output monitor (USCOM) uses continuous-wave Doppler ultrasound technology directed at aortic or pulmonary valve flow to calculate CO and systemic vascular resistance (SVR) and can be used in children with shock (22). While these non-invasive methods can be used to augment physiological outputs, they do not provide insight into the underlying pathobiology. The findings described herein raise the hypothesis that the use of non-invasive LDPMI for assessing vascular reactivity may be used to augment monitoring during critical illness. In this regard, use of LDPMI in sepsis could be analogous to the non-invasive assessment of the healing potential of burn wounds using a hand-held thermal imager, which has been shown to have good validity in assessing outcomes (23, 24).

It should be noted that while this case series only includes pediatric survivors who had resolution of their septic shock within 14 days of hospital admission, all patients had vastly different severities of illness, pathogens and underlying co-morbidities highlighting the heterogeneity of pediatric sepsis. Yet despite this heterogeneity, the endothelial dysfunction observed was strikingly similar in its temporal variation. One could postulate that these similar temporal responses to ACh are only true for patients from whom recovery and survival is expected. This would be opposed to patients who have early or late mortality from sepsis. With regards to the latter, a notable proportion of adult patients with sepsis develop persistent inflammation, immunosuppression, and catabolism syndrome (PICS), which can also occur in critically ill pediatric patients (25, 26). PICS is characterized by a vicious cycle of persistent inflammation, organ injury, loss of muscle mass, and emergency myelopoiesis simultaneously resulting in overproduction of immunosuppressive myeloid-derived suppressor cells (MDSCs), anemia, and lymphopenia (25). In patients meeting these criteria who remain critically ill after 14 days, mortality is high, and many patients develop recurrent infections (25). Considering the detrimental effects of PICS on organ function, it seems conceivable that patients with PICS may suffer from persistent vascular dysfunction as well. LDPMI-based assessments of vascular function over the course of hospitalization could potentially help predict which patients might develop PICS, though this is theoretical. Beyond predictions of hospital course, this method could potentially also be used to monitor patient recovery in terms of vascular health after discharge from a septic shock admission. For instance, patients could be followed in an outpatient setting where vascular reactivity can be monitored non-invasively using LDPMI-based assessments. In doing so, both the likelihood of recovery as well as the trajectory of recovery may potentially be surveilled. In relation to the case series presented, the patient with the worst ACh response by Day 14 (Case 3) later went on to develop secondary fungemia as well as require antihypertensive therapies. While this single instance should not be interpreted as cause and effect, use of such devices to prospectively predict peripheral artery disease in adults has been utilized and one could conceive a similar application in pediatrics during and after critical illness (27).

Beyond the small number of patients in the series, a significant limitation noted is the absence of baseline LDPMI-based vascular response assessments. As such, for the patients included in this series, it is not known how LDPMI assessments to ACh and SNP during septic shock compare to LDPMI assessments prior to the onset of illness. Furthermore, whether or not the patients included in this study will have persistent vascular dysfunction as measured by LDPMI after 14 days is unknown. In addition, as this is a case series and owing to the relatively high survival and limited duration of illness in pediatric sepsis, the number of patients who were available to have 14 day measurements was limited. Lastly, it is appreciated that the statistical analyses included here, although significant for some measurements, include a limited number of patients and should thus be interpreted in that context. Therefore, it would be of interest to interrogate how temporal alterations in vascular function during septic shock compare across a larger cohort of patients who could complete more comprehensive longitudinal assessments.

Overall, the present study provides preliminary insights into the temporal alterations in vascular function during septic shock as measured by non-invasive LDPMI assessments in pediatric patients. Utilization of this method may potentially provide additional, temporal insights into the underlying pathology of the vasculature and predict cardiovascular risk factors in pediatric patients with septic shock.

## Data Availability Statement

The original contributions presented in the study are included in the article/supplementary material, further inquiries can be directed to the corresponding author/s.

## Ethics Statement

The studies involving human participants were reviewed and approved by the Institutional Review Board at Vanderbilt University Medical Center (IRB# 200418). Written informed consent to participate in this study was provided by the participants' legal guardian/next of kin.

## Author contributions

RS designed the case series and performed data collection and statistical analyses. CW and RS wrote sections of the manuscript. All authors contributed to manuscript revision, read, and approved the submitted version.

## Funding

The study was supported by a grant from NIH NIGMS (R35 GM138191) to RS.

## Conflict of interest

The authors declare that the research was conducted in the absence of any commercial or financial relationships that could be construed as a potential conflict of interest.

## Publisher's note

All claims expressed in this article are solely those of the authors and do not necessarily represent those of their affiliated organizations, or those of the publisher, the editors and the reviewers. Any product that may be evaluated in this article, or claim that may be made by its manufacturer, is not guaranteed or endorsed by the publisher.
